# Evidence That Parietal Lobe Fatty Acids May Be More Profoundly Affected in Moderate Alzheimer’s Disease (AD) Pathology Than in Severe AD Pathology

**DOI:** 10.3390/metabo8040069

**Published:** 2018-10-26

**Authors:** Muhammad L. Nasaruddin, Xiaobei Pan, Bernadette McGuinness, Peter Passmore, Patrick G. Kehoe, Christian Hölscher, Stewart F. Graham, Brian D. Green

**Affiliations:** 1 Institute for Global Food Security (IGFS), Queen’s University Belfast, Stranmillis Road, Belfast BT9 6AG, Ireland; mbinnasaruddin01@qub.ac.uk (M.L.N.); x.pan@qub.ac.uk (X.P.); 2 Centre for Public Health, School of Medicine, Dentistry and Biomedical Sciences, Queen’s University, Belfast BT12 6BA, Ireland; B.McGuinness@qub.ac.uk (B.M.); P.Passmore@qub.ac.uk (P.P.); 3 Dementia Research Group, Institute of Clinical Neurosciences, School of Clinical Sciences, University of Bristol, Bristol BS10 5NB, UK; Patrick.Kehoe@bristol.ac.uk; 4 Research and Experimental Center, Henan University of Traditional Chinese Medicine, Longzihu University Campus, 156 Jinshui Dong Road, Zhengzhou 450000, China; christian_holscher@me.com; 5 Metabolomics Research, Beaumont Research Institute 3811 W. 13 Mile Road, Royal Oak, MI 48073, USA; Stewart.Graham@beaumont.org; 6 Metabolomics Research, Oakland University-William Beaumont School of Medicine, Rochester, MI 48309, USA

**Keywords:** fatty acid, GC-MS, Alzheimer’s disease, dementia with Lewy bodies, metabolomics, lipidomics

## Abstract

Brain is a lipid-rich tissue, and fatty acids (FAs) play a crucial role in brain function, including neuronal cell growth and development. This study used GC-MS to survey all detectable FAs in the human parietal cortex (Brodmann area 7). These FAs were accurately quantified in 27 cognitively normal age-matched controls, 16 cases of moderate Alzheimer’s disease (AD), 30 severe AD, and 14 dementia with Lewy bodies (DLB). A total of 24 FA species were identified. Multiple comparison procedures, using stepdown permutation tests, noted higher levels of 13 FAs but the majority of changes were in moderate AD and DLB, rather than severe AD. Subjects with moderate AD and DLB pathology exhibited significantly higher levels of a number of FAs (13 FAs and 12 FAs, respectively). These included nervonic, lignoceric, *cis*-13,16-docosadienoic, arachidonic, *cis*-11,14,17-eicosatrienoic, erucic, behenic, α-linolenic, stearic, oleic, *cis*-10-heptanoic, and palmitic acids. The similarities between moderate AD and DLB were quite striking—arachidic acid was the only FA which was higher in moderate AD than control, and was not similarly affected in DLB. Furthermore, there were no significant differences between moderate AD and DLB. The associations between each FA and a number of variables, including diagnosis, age, gender, Aβ plaque load, tau load, and frontal tissue pH, were also investigated. To conclude, the development of AD or DLB pathology affects brain FA composition but, intriguingly, moderate AD neuropathology impacts this to a much greater extent. Post-mortem delay is a potential confounding factor, but the findings here suggest that there could be a more dynamic metabolic response in the earlier stages of the disease pathology.

## 1. Introduction

Alzheimer’s disease (AD) is a neurodegenerative disorder characterized by progressive memory loss and deteriorating cognitive abilities in older populations. Dementia currently affects around 820,000 people in the United Kingdom, and the estimated cost of the condition to the economy is £24 billion per annum [1]. Global projections suggest that the number of people affected by AD will triple to 115 million by the year 2050 [1]. The main pathological hallmarks of AD are the accumulation of β-amyloid plaques and neurofibrillary tangles [2,3]. There has been a concerted effort to investigate these two histological features, but the exact causes and pathological consequences of AD remain to be elucidated.

Metabolomics is the investigation of small, often chemically diverse molecules, including lipids, saccharides, steroids, bile acids, and amino acids [4,5]. Lipidomics is a subdiscipline of metabolomics focusing entirely on the measurement of lipid and lipid-like molecules. Profiling of the lipidome has revealed alterations in (i) lipid metabolism [4,6,7]; (ii) lipid-mediated signaling processes [4,8,9]; and (iii) biochemical interactions with other lipids, proteins, and metabolites [10,11]. Ultimately, these techniques could lead to new discoveries in terms of disease pathogenesis and pharmacological targets [12,13]. Lipid profiling techniques are increasingly being employed in AD in the hope that they might provide new mechanistic insights or novel diagnostic biomarkers [4]. The most common approach has been the profiling of fatty acids (FAs) by either GC-MS or LC-MS. There are clear reasons for applying precise lipid profiling techniques in an AD context. The brain is one of the most lipid-rich tissues in the human body [14] and, also, the earliest reports of AD pathology noted the occurrence of “fat inclusions” or “lipid granules” in the brain. Around 50% of neuronal cell membranes are composed of fatty acids (FA) that are polyunsaturated [15]. These FAs appear to be incorporated into membrane phospholipids and secondary signaling messengers, which modulate oxidative stress and inflammatory processes in neurons [15].

It is surprising that FA profiling techniques have shown relatively subtle changes in brain FA composition [16]. This, in part, can be attributed to current technical limitations, which makes it difficult to detect changes in the subcellular distribution of FAs. However, it is clear that the brain concentrations of a small number of FAs are affected by the development of AD pathology [16]. Altered levels of stearic acid, arachidonic acid, and oleic acid have been observed in the frontal and temporal cortex of human cases of AD [16]. This investigation also showed that the parietal cortex is comparably much less affected [16]. Currently, it is a major challenge to distinguish between FA alterations that are secondary to the development of AD, and those that which may contribute to the disease process. Progress in this area is hampered by the fact that most studies (including our own) have not specifically determined how the severity of AD pathology affects the FA profile [12,13,16,17]. There is a need for more research in this area. Recent metabolomic studies profiling APP/PS1 mice found that metabolites, including many lipid species, are affected by the development of AD pathology. However, the disturbances are transient in nature, not occurring in either a persistent or progressive manner [18]. Lipid disturbances in both brain and plasma appear to be highly dependent on the disease stage/severity, and many lipids differ much less in the later stages of the pathology [18].

This study investigated how exactly human brain FA profiles are affected by the extent of neuropathological change. The parietal cortex was selected because it is not the primary site of AD pathology and, also, because FA composition is largely unaffected in AD. We used a fatty acid methyl ester (FAME) methodology to survey all detectable FA species present in this brain region. A quantitative method was established for 24 of these. A full range of analytical FAME standards and internal standards were employed to quantify FAs in pathologically and clinically confirmed cases of AD and dementia with Lewy bodies (DLB), in addition to normal age-matched control cases. To assess the influence of AD severity on FAs, the cases of AD were classified as either moderate (“intermediate” AD neuropathological change, Braak tangle stage III–IV) or severe (“high” AD neuropathological change, Braak tangle stage V–VI).

## 2. Results

**Figure 1:**
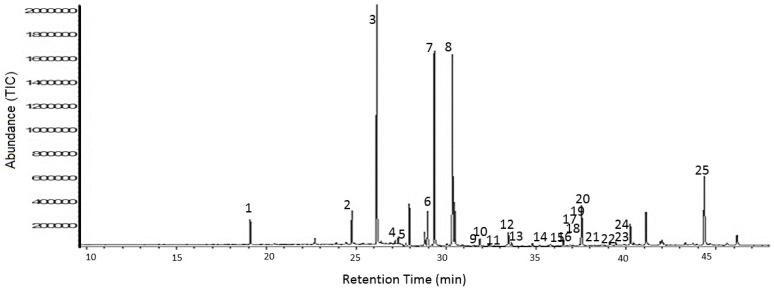
GC-MS detection of brain fatty acids. A typical total ion chromatogram (TIC) of fatty acid methyl esters (FAMEs) from post-mortem brain tissue. The peaks were identified as 1. lauric acid, 2. pentadecanoic acid, 3. palmitic acid, 4. palmitoleic acid, 5. heptadecanoic acid, 6. *cis*-10-heptadecanoic acid, 7. stearic acid, 8. oleic acid, 9. linolelaidic acid, 10. linoleic acid, 11. arachidic acid, 12. *cis*-11-eicosanoic acid, 13. linolenic acid, 14. heneicosanoic acid, 15. *cis*-8,11,14-eicosatrienoic acid, 16. erucic acid, 17. *cis*-11,14,17-eicosatrienoic acid, 18. *cis*-11,14-eicosadienoic acid, 19. behenic acid, 20. arachidonic acid, 21. tricosanoic acid, 22. *cis*-13,16-docosadienoic acid, 23. lignoceric acid, 24. nervonic acid, 25. docosahexaenoic acid.

**Figure 2:**
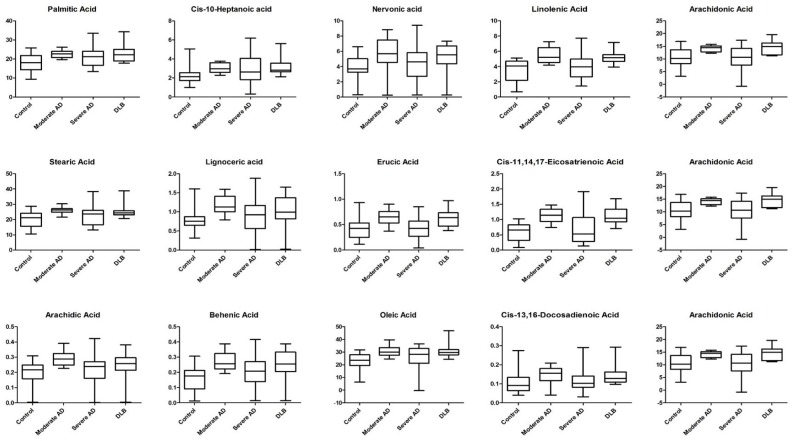
Brain fatty acids (g/kg) significantly altered across moderate Alzheimer’s disease (AD), severe AD, and dementia with Lewy bodies (DLB) groups. A total of 13 brain fatty acid (FA) species were found to be significant across the 4 groups. FA species concentrations are displayed as box-and-whisker plots with maximum and minimum values.

Initial survey of the FA composition of human post-mortem parietal cortex identified 24 quantifiable (calibration curves for FAMEs with *R*^2^ values of <0.98 were deemed inadmissible) individual FAME peaks that were consistently present in all subject groups. The potential for there to be differences between subject groups was immediately evident from relative peak intensity data. FAME peak identities were assigned by mass spectrometry and subsequently confirmed using FAME analytical standards. Typical total ion chromatograms (TICs) for specimens from cases of moderate and severe AD, DLB, and age-matched control subjects, are shown in Figure 1. Initially the Kruskal–Wallis test was used to shortlist those FAs presenting at least one statistically significant difference between any of the 4 subgroups (*p* < 0.05; FDR < 0.05) (Table 1). A total of 13 FAs differed, including (i) 4 saturated FAs: lignoceric acid (C24:0), behenic acid (C22:0), arachidic acid (C20:0), and stearic acid (C18:0); (ii) 5 monounsaturated FAs: nervonic acid (C24:1Δ15), oleic acid (c-C18:1Δ9), *cis*-10-heptadecanoic acid (C17:1Δ10), palmitic (c-C18:1Δ9), and erucic acid (C22:1Δ130); and (iii) 4 polyunsaturated FAs; *cis*-13,16-docosadienoic acid (C22:2Δ13,16), arachidonic acid (C20:4Δ5,8,11,14), *cis*-11,14,17-eicosatrienoic acid (C20:3Δ11,14,17), and α-linolenic acid (C18:3Δ9,12,15) (Table 1). Multiple comparison procedures using stepdown permutation *p*-values, on the ranked data, were used to investigate the nature of group differences for variables, with a false discovery rate of 0.05 or less (Table 2). This revealed that only one FA (oleic acid (c-C18:1Δ9)) significantly differed between severe AD and control samples (Table 2). Contrastingly, a total of 12 and 13 FAs were significantly (*p* < 0.05) elevated in DLB and moderate AD, respectively, compared with control. The levels of 5 FAs differed between severe AD and DLB: arachidonic acid (C20:4Δ5,8,11,14), *cis*-11,14,17-eicosatrienoic acid (C20:3Δ11,14,17), erucic acid (C22:1Δ130), behenic acid (C22:0), and linolenic acid (C18:3Δ9,12,15) (Table 2). The profile of moderate and severe AD samples differed markedly, with 9 FAs being significantly lower in severe AD (Figure 2). Significant differences were found for Total FA, Total SFA, Total monounsaturated FA (MUFA), Total polyunsaturated FA (PUFA), but not for either Omega 3/Omega 6 ratio or 16:1/16:0 ratio (Table S2). No significant associations were detected between any FA and the subject age, frontal tissue pH, and beta-amyloid levels (Table S3). Only linoleic acid differed between male and female subjects (41% higher in females). Six FAs (lignoceric acid, arachidonic acid, *cis*-11,14,17-eicosatrienoic acid, linolenic acid, arachidic acid, and stearic acid) negatively correlated with post-mortem delay. Three FAs (*cis*-11,14,17-eicosatrienoic acid, erucic acid, and linoelaidic acid) negatively correlated with levels of tau protein.

The scores plot generated by unsupervised principal component analysis (PCA) showed only very weak separation between the 4 groups (Figure S1). The PCA results showed that the first component (PC1) explained 36.3% of the variation. Although there was overlap among the 4 groups, it was noteworthy that moderate AD and DLB cases were distributed at the extreme end of PC1.

## 3. Discussion

This study accurately quantified the FA composition of post-mortem human brain tissue from 87 subjects: control subjects (*n* = 27); moderate AD (*n* = 16), severe AD (*n* = 30), and DLB (*n* = 14). A key strength of this study is its focused approach to brain FA measurements. It represents the widest brain FA coverage measured, thus far, in AD or other forms of dementia. Previous studies have found gender-related differences in brain FA content in AD [12], but this was not evident in the present study. Other studies typically measure a much narrower range of FAs, of perhaps 14 or 16 FAs at most [16,19]. We compared the measured brain FA levels as percentage % of total FA content, and found them to be broadly comparable with a number of other studies [16,17,20,21,22,23,24,25,26]. In general, the findings indicate that, in parietal cortex, there is a trend for higher FA levels in moderate AD, but not in cases of severe AD. Several FAs where significantly affected, including lignoceric acid, *cis*-13,16-docosadienoic, arachidonic acid, *cis*-11,14,17-eicosatrienoic acid, erucic acid, linolenic acid, and stearic acid. This is not the first time that FA concentrations have been shown to be higher in AD [20], however, these earlier changes suggest mechanisms involving lipid metabolism in response to the development of disease pathology. It has previously been suggested that the relatively higher brain FA content may be due to ceramide accumulation due to the activation of sphingomyelinase in oligodendrocytes, induced by increases in amyloid beta peptide levels [20]. 

A particular limitation of the present study was that post-mortem delay was significantly longer in cases of severe AD, compared with cases of moderate AD. In general, the post-mortem delay durations for the brain specimens obtained were relatively long. In our tissue requests, we did our best to obtain samples with as short a delay as possible, to minimize tissue degradation/oxidation which, in turn, could affect the FA composition. Where possible, we attempted to match groups as much as possible with respect to post-mortem delay. This was performed within the constraints of the available tissue with the appropriate pathological characteristics. It is possible that post-mortem delay is a confounding factor which explains significant differences for some FAs between Severe and Moderate AD groups. An in-depth analysis finds that there is some support for this—there were modest but significant associations between the duration of post-mortem delay and concentrations of six individual FAs. All of these were negative associations, suggesting that longer post-mortem delay may decrease these FAs. Five of the six FAs where among the 13 shortlisted as differing between groups. Furthermore, it is also clear that there were group differences as far as aggregated FA levels are concerned (total FA, total saturated, total monounsaturated, or total polyunsaturated). For this reason, the findings here should be interpreted with a degree of care because post-mortem delay is an uncontrolled variable.

Nervonic acid is a product of the desaturation and elongation processes of several fatty acids, including palmitic acid, stearic acid, and oleic acid. Through the action of stearoyl CoA desaturase (SCD) enzyme, these fatty acids undergo a series of elongation steps prior to the production of nervonic acid. Higher concentrations of these fatty acids, in moderate AD pathology, could be attributed to the increased activity of SCD itself. A recent study has demonstrated that nervonic acid and several mono-unsaturated fatty acids produced by SCD are markedly upregulated in brain samples of AD patients [27]. Higher FA levels and SCD activity have been shown to correlate closely with cognitive impairment [27]. Although our findings contrast with that of Astarita et al. [27], SCD activity does provide a potential pathogenic mechanism. 

Higher palmitic acid concentrations in moderate AD pathology indicate damaging effects on the brain. Palmitic acid has been shown to induce tau hyperphosphorylation and to elevate β-secretase activity in embryonic rat cortex cultures [28]. Furthermore, the trend for palmitic acid is in keeping with the plasma levels observed in another study [19]. The case is also similar for oleic and stearic acid plasma levels in AD patients recently reported in a longitudinal population-based study by Ronnema et al. (2012) [29]. It is interesting to note that only oleic acid was found to be significantly elevated when Control was paired with Severe AD (Figure 2). Another study reported similar finding, but uniquely in the white brain matter of AD patients [17].

Omega-3 and -6 (*n*-3/*n*-6) fatty acids are referred to as essential fatty acids. synthesized from dietary linolenic or linoleic acids through series of saturation and elongation reactions [30]. Both *n*-3 and *n*-6 fatty acids have been implicated in the modulation of brain inflammatory processes [30,31,32]. Fatty acids, such as arachidonic acid (AA), eicosapentanoic acid (EPA; C20:5), and DHA are thought to modulate the severity and duration of AD inflammatory processes [33,34]. These fatty acids exert their inflammatory function through their conversion to potent eicosanoids, such as prostaglandins, thromboxanes, and leukotrienes (by AA), or resolvins and docosatrienes (by EPA and DHA) by means of cyclooxygenase (COX) and/or lipoxygenase (LOX) enzymes [29,35]. 

In the present study, DHA did not differ between any of the 4 groups, a finding which is consistent with several previous studies [16,17,36]. Other reports, on the other hand, showed lower levels in AD brain subjects [13,19,21,22,37]. Snowden and colleagues found DHA levels in individuals with significant AD neuropathology to be unchanged in both the inferior temporal gyrus and the cerebellum, but it was elevated in middle frontal gyrus [38]. For linolenic acid (a precursor of DHA as well as EPA) however, we did detect higher levels in moderate AD. Linolenic acid is an omega-3 FA with anti-inflammatory activity [37,38]. The elevated linolenic acid (in moderate AD) observed, here, may constitute an anti-inflammatory to the development of early AD disease pathology. Interestingly, arachidonic acid was higher in moderate AD. Eicosanoids derived from arachidonic acid are typically pro-inflammatory agents. It has been demonstrated that arachidonic acid is converted by the enzymatic activity of COX and LOX on increased levels of pro-inflammatory cytokines and activation of neutrophils, resulting from eicosanoid biosynthesis from enzymatic activity of COX and LOX on arachidonic acid [39]. Our study showed decreased levels of both linolenic acid and arachidonic acid (Figure 2). While the reason for such occurrences in the brain are yet to be fully determined, the trend, however, was found to be in keeping with plasma levels of recently studied AD patients [40]. The decreasing trend of AA and LA seen in plasma AD patients was indicative of a deficiency in neuroprotective elements against the pathology of the disease, and the excessive inflammation that was occurring [40]. Furthermore, the significantly higher *cis*-13,16-docosadienoic concentrations in moderate AD group could form part of an anti-inflammatory response to the development of AD. Higher levels of *cis*-13,16-docosadienoic concentrations could provide neuroprotection by blockading COX enzyme activity [34]. The overlapping levels of fatty acids seen and described earlier in age-matched Controls with moderate and severe AD subjects, provide a unique profile which may serve as indicating potential biomarkers of the disease.

Dementia with Lewy bodies (DLB) is the second major type of senile, degenerative dementia after AD. It is characterized by the presence of cytoplasmic inclusions of highly conserved amyloidogenic α-synuclein [41] protein that mirrors tau proteins in AD. It is localized, in part, to presynaptic terminals where it loosely associates with synaptic vesicles [42,43]. Numerous studies have been conducted on its structural organization at various stages of fibrillation and inclusion formation. 

Fatty acids have been found to play an important role in the conversion of normal, soluble α-synuclein to insoluble and potentially cytotoxic forms. In vitro studies of mouse embryonic stem cells (MES) neurons showed PUFA promotes the appearance of oligomeric forms of α-synuclein in a time-dependent manner [44], while increasing the degree of unsaturation of fatty acids strikingly enhanced the amount of soluble forms [44]. It is further noted that higher levels of α-synuclein in DLB brains may be linked to the changes in the composition of endogenous brain fatty acid species. Sharon et al. (2003) reported a consistently lower linolenic acid and higher DHA levels in frontal cortex of DLB brains [45]. In contrast to their findings, our results indicate higher concentrations of linolenic acid when DLB are either paired with Control or severe AD groups (Figure 2; Table 2).

In addition, the study also found no changes or significant alterations in arachidonic acid levels, as well as other MUFAs [45]. We, however, saw higher concentrations of such species in both Control, and few in severe AD-paired groups. Furthermore, the higher abundance of saturated fatty acids, including palmitic acid, stearic acid, behenic acid, and lignoceric acid, observed in our study, may suggest a protective mechanism by counterbalancing the oligomeric formation of α-synuclein by eicosatrienoic, linolenic, and arachidonic acid in DLB brains (Figure 2; Table 2). One study demonstrated the impact of DHA and arachidonic acids imposing conformational changes in the structure of α-synuclein, whereas the action of palmitic and arachidic acids remained unchanged [46]. To the best of the author’s knowledge, this is the first study that directly compares brain fatty acid profiles of Alzheimer’s disease and DLB. Despite the fact that the overlapping FA species between severe AD and Control with DLB are relatively unknown, with regards to their individual function, it still highlights the probability of such fatty acids being used as potential biomarkers to distinguish the differences between these groups. Principal component acids in both healthy control, moderate, and severe AD groups could serve as potential biomarkers that warranted further functional studies. Particular attention, in future, should be paid to controlling the controlling of post-mortem delay. While most FAs mentioned above played intrinsic roles in the inflammatory/anti-inflammatory processes of AD, some remained to be understood, given the discrepancies and limited data available in the current literature. The FA profiles of AD and DLB are very similar, perhaps reflecting common progression of the disease, but conclusions such as this require additional studies. This paper provides an insight into the changes of FA metabolism in the development of AD, as well differences that can be observed in the profiles of DLB subjects. 

## 4. Materials and Methods 

### 4.1. Reagents and Analytical Standards

The following fatty acid and fatty acid methyl ester standards were purchased from Sigma Aldrich (Gillingham, Dorset, UK): tricosanoic acid (C23:0), behenic acid (C22:0), *cis*-11,14-eicosadienoic acid (C20:2 Δ11,14), heptadecanoic acid (C17:0), *cis*-10-pentadecanoic acid (C15:1Δ10), pentadecanoic acid methyl ester (C15:0) (PDA), palmitic acid methyl ester (C16:0), palmitoleic acid methyl ester (C16:1Δ9), *cis*-10-heptadecanoic acid methyl ester (C17:1Δ10), stearic acid methyl ester (C18:0), elaidic acid methyl ester (t-C18:1Δ9), oleic acid methyl ester (C18:1Δ9), linolelaidic acid methyl ester (t-C18:2 Δ9, 12) linoleic acid methyl ester (C18:2 Δ9,12), arachidic acid methyl ester (C20:0), linolenic acid methyl ester (C18:3 Δ9,12,15), heneicosanoic acid methyl ester (C21:0), *all-cis*-11,14,17-eicosatrienoic acid methyl ester (C20:3 Δ11,14,17), arachidonic acid methyl ester (C20:4 Δ5,8,11,14), *all-cis*-13,16-docosadienoic acid methyl ester (C22:2 Δ13,16), lignoceric acid methyl ester (C24:0), nervonic acid methyl ester (C21:1 Δ15), docosahexanoic acid methyl ester (C22:6 Δ4,7,10,13,16,19) (DHA), *all-cis*-8,11,14-eicosaterinoic acid methyl ester (C20:3 Δ8,11,14), erucic acid methyl ester (C22:1 Δ13). All solvents used (methanol *n*-hexane and dichloromethane) were CHROMASOLV HPLC grade (Sigma Aldrich, UK). Hydrogen chloride (1.25 M) in methanol was purchased from Fluka Analytical (UK). 

### 4.2. Human Post-Mortem Tissue

As previously described by Graham et al. (2014) [47], post-mortem tissue samples (parietal neocortex, Brodmann area 7) were obtained from pathologically and clinically confirmed cases of AD (*n* = 46), DLB (*n* = 14), and normal age-matched controls (*n* = 27) [47] (Table S1). The parietal cortex (Brodmann 7) region was selected as it not the primary site of AD pathology. Studies have shown that FA composition of the parietal cortex is less affected than other brain regions, such as the frontal or temporal cortex [16]. The AD was classified as moderate (“intermediate” AD neuropathological change [48], and also Braak tangle stage III–IV; *n* = 16) or severe (“high” AD neuropathological change [48], and also Braak tangle stage V–VI; *n* = 30). Mixed pathology cases were excluded from the study. Cases were geographically spread across the United Kingdom (Bristol, Newcastle, and London) and were obtained through the Brains for Dementia Research (BDR; see acknowledgements). Sample selection did not control for post-mortem delay, and there was a significantly difference between Severe AD and Moderate AD (*p* = 0.03). The neuropathological diagnoses were made using widely accepted criteria [48,49], uniformly applied according to a standardized protocol by members of the BDR Neuropathology Group. DLB was diagnosed on the basis that (i) there was a clinical diagnosis of dementia, (ii) some Lewy bodies were present in the neocortex [50]. Consent and ethical approval for the use of tissue were obtained by individual brain banks, all of which are licensed by the Human Tissue Authority (UK). Consent and ethical approval for the use of tissue was obtained by individual brain banks, all of which are licensed by the Human Tissue Authority. The study was conducted in accordance with the Declaration of Helsinki, and the protocol was reviewed by the School Research Ethics Committee (Biological Sciences, Queen’s University Belfast) (0512-GrahamS).

### 4.3. Sample Preparation and GC-MS Analysis 

Frozen tissue samples stored at −80 °C were initially lyophilized by placing them in a freeze-drier at −50 °C. Following the complete removal of moisture tissue specimens, they were placed into a cryogenic grinder (Model 6850, SPEX SamplePREP, UK), which uses liquid nitrogen as a coolant throughout the milling process and, thus, avoids heat generation which could affect the concentration of metabolites in the brain tissue samples. Once milled, all samples were stored at −80 °C. Lipids were extracted from tissue by the Folch extraction method [51]. Briefly, 50 mg of powder (weighed out into sterile Eppendorf tubes (2 mL) chilled on ice). Methanol/water (50% v/v; 1 mL) was added to each sample and shaken for 10 min using a Minimix Standard Shaker (Merris Engineering, Maidenhead, Berkshire). Samples were then sonicated for 15 min. Protein was removed by centrifugation at 16,000× *g* at 4 °C for 20 min. To assess the recoveries of FA species, lauric acid (C12:0) was selected as an internal standard, as it was found to be completely absent in all the post-mortem human brain samples. Lauric acid was added to each sample pellet (100 ng/μL) and FAs were extracted in 1 mL of DCM, transferred to sterile test tubes, and evaporated to dryness under nitrogen. FAs were derivatized to fatty acid methyl esters (FAMEs) by reconstituting the dried extracts in 2 mL hydrogen chloride in methanol. Samples were subsequently cooled, and 1 mL of water was added. The FAMEs were extracted in 1 mL of hexane and subsequently analyzed using an Agilent GC (model 7890, Wilmington, DE, USA) coupled to an MS detector (Agilent model 5975C, Wilmington, DE, USA). The FAMEs procedure creates methyl esters from free fatty acids, but also from esterified fatty acids and, therefore, measurements reflect the total levels of unesterified and esterified fatty acids.

Samples were injected (inlet temp 220 °C, split-mode ration of 15:1) onto a CP-Sil88 fused silica capillary column (100 m × 0.25 mm × 0.25 5 μm) (Agilent, UK) with helium as the carrier gas at a constant flow of 1 mL/min. The initial temperature gradient began at 100 °C, increasing at 4 °C/min to 220 °C/min, and held for 5 min. Following this, the gradient increased at 4 °C/min to 240 °C/min, and was held for 8 min. The mass selective detector (MSD) operated at 70 eV in dual scan/single ion monitoring (SIM) mode; source temp 230 °C, quad temp 150 °C and the interface temp 225 °C. The full scan ranged from *m/z* 50 to 550, whilst SIM mode targeted the molecular ion and another appropriate ion selected from the fragmentation pattern, each ion having a dwell time of 100 ms. All FAMEs were confirmed using purchased analytical-grade standards. Quantification was based on a linear regression model formed from a five-point calibration curve from the individual FAMEs, which were acquired at a low (0–20 ng/mL) or high concentration range (0–300 ng/mL). FFA concentrations are reported as g/kg post-mortem human brain dry weight, and corrected to the internal standard. Fatty acids with calibration curves (*n* = 3) of poor linearity of (*R*^2^ < 0.9) were not quantified

### 4.4. Data Analysis

Normality of distribution was assessed using the Anderson–Darling test. Nonparametric methods were used since the assumption that FA concentrations were normally distributed was not satisfied for many variables. The four groups were compared on metabolite levels using Kruskal–Wallis tests. Elaidic and cis-10-pentadeconaic acid were excluded, given the large prevalence of values of 0. Given the large number of variables tested, false discovery rates were computed based on the *p*-values from the Kruskal–Wallis tests on the remaining 24 variables. Multiple comparison procedures using stepdown permutation *p*-values, on the ranked data, were used to investigate group differences for variables with a false discovery rate of 0.05 or less. Statistical analysis used the SAS System for Windows version 9.3 (Cary, NC, USA). All tests were two-sided, and *p* < 0.05 was considered statistically significant. Graphical representations of the data were produced using Prism (GraphPad 5.0, La Jolla, CA, USA). Group comparisons of patient characteristics (age, % female, and post-mortem delay) were compared by parametric one-way ANOVA. Associations between FAs and continuous variables (age, post-mortem delay, beta-amyloid, or tau) were tested by Spearman’s rank correlation coefficient. The relationship between FA levels and gender were carried out by dividing subjects into male and female and conducting a Mann–Whitney *U t*-test for each FA. PCA was completed using Simca P (v14.1; Umetrics, Umea, Sweden) using mean centered and log transformed data.

## Figures and Tables

**Table:** 

Fatty Acid				Control				Moderate AD			
Name	Lipid No.	RT	Mass	Mean ± SD	Q1	Median	Q3	Mean ± SD	Q1	Median	Q3
**Docosahexanoic Acid**	C22:6Δ4,7,10,13,16,19	45.01	342.51	24.77 ± 6.695	18.830	24.640	30.200	30.184 ± 4.215	27.600	28.540	33.130
**Nervonic Acid**	C24:1Δ15	40.68	380.65	3.925 ± 1.527	3.331	3.686	5.014	5.517 ± 2.549	4.795	5.706	7.311
**Lignoceric Acid**	C24:0	39.55	382.66	0.767 ± 0.317	0.657	0.756	0.851	1.187 ± 0.256	1.003	1.126	1.404
***cis*-13,16-Docosadienoic acid**	C22:2Δ13,16	38.89	350.58	0.103 ± 0.052	0.065	0.091	0.125	0.146 ± 0.044	0.122	0.156	0.178
**Arachidonic acid**	C20:4Δ5,8,11,14	38.08	318.49	10.81 ± 3.309	8.280	10.270	13.600	14.04 ± 1.244	12.950	14.480	15.050
**Tricosanoic Acid**	C23:0	37.42	368.64	0.165 ± 0.140	0.024	0.138	0.248	0.218 ± 0.207	0.014	0.285	0.417
***cis*-11,14,17-Eicosatrienoic acid**	C20:3Δ11,14,17	28.93	320.51	0.565 ± 0.292	0.322	0.656	0.807	1.098 ± 0.242	0.937	1.139	1.294
**Erucic acid**	C22:1Δ13	35.60	352.59	0.409 ± 0.188	0.260	0.427	0.514	0.627 ± 0.145	0.539	0.653	0.720
***cis*-8,11,14-Eicosatreinoic acid**	C20:3Δ8,11,14	37.05	320.51	1.010 ± 0.389	0.729	1.031	1.245	1.253 ± 0.523	1.262	1.295	1.519
**Behenic acid**	C22:0	35.18	354.61	0.160 ± 0.085	0.095	0.177	0.211	0.273 ± 0.060	0.222	0.258	0.323
***cis*-11,14-Eicosadienoic acid**	C20:2 Δ11,14	35.18	308.50	0.549 ± 0.482	0.253	0.357	0.629	0.534 ± 0.411	0.242	0.449	0.648
**Heneicosanoic acid**	C21:0	34.39	340.58	0.018 ± 0.007	0.011	0.020	0.024	0.026 ± 0.009	0.022	0.026	0.030
**Linolenic acid**	C18:3Δ9,12,15	34.20	294.26	3.514 ± 1.315	2.469	4.067	4.613	5.401 ± 1.048	4.564	5.209	6.059
***cis*-11-Eicosanoic acid**	C20:1 Δ11	33.45	310.50	2.010 ± 0.803	1.539	2.016	2.597	2.616 ± 1.266	2.075	2.905	3.513
**Arachidic acid**	C20:0	32.85	326.56	0.200 ± 0.070	0.165	0.216	0.247	0.292 ± 0.047	0.259	0.288	0.315
**Linoleic acid**	c-C18:2Δ9,12	32.38	294.47	0.702 ± 0.478	0.386	0.777	1.012	0.941 ± 0.593	0.751	0.989	1.327
**Linolelaidic acid**	t-C18:2Δ9,12	31.29	294.47	0.019 ± 0.009	0.012	0.016	0.024	0.023 ± 0.011	0.014	0.023	0.029
**Oleic acid**	c-C18:1Δ9	30.82	297.49	22.92 ± 6.249	19.400	23.560	27.370	30.664 ± 4.219	27.490	29.960	33.460
**Stearic acid**	C18:0	29.79	298.50	20.37 ± 5.037	15.730	21.290	23.950	25.985 ± 2.407	24.770	26.170	26.770
***cis*-10-Heptadecanoic acid**	C17:1Δ10	29.30	284.48	2.275 ± 0.918	1.720	2.144	2.537	3.054 ± 0.485	2.631	2.965	3.532
**Heptadecanoic acid**	C17:0	28.21	283.48	0.308 ± 0.104	0.254	0.300	0.366	0.366 ± 0.035	0.344	0.372	0.396
**Palmitoleic acid**	C16:1Δ9	27.77	270.45	0.842 ± 0.322	0.653	0.790	1.062	1.046 ± 0.221	0.928	1.113	1.186
**Palmitic acid**	C16:0	26.60	270.45	18.09 ± 4.308	14.450	18.110	21.500	22.670 ± 1.848	21.080	22.680	23.820
**Pentadecanoic acid**	C15:0	24.91	256.42	0.159 ± 0.061	0.118	0.165	0.184	0.174 ± 0.028	0.154	0.184	0.192

**Table:** 

Fatty Acid				Severe AD				DLB			*p*-Value		
Name	Lipid No.	RT	Mass	Mean ± SD	Q1	Median	Q3	Mean ± SD	Q1	Median	Q3	KW	FDR
**Docosahexanoic Acid**	C22:6Δ4,7,10,13,16,19	45.01	342.51	27.694 ± 9.311	20.090	26.970	34.740	32.329 ± 9.102	26.010	31.960	36.140	0.0328	0.0525
**Nervonic Acid**	C24:1Δ15	40.68	380.65	4.352 ± 2.385	2.747	4.633	5.775	5.348 ± 2.763	4.417	5.547	6.530	DLB	0.0251
**Lignoceric Acid**	C24:0	39.55	382.66	0.942 ± 0.442	0.576	0.924	1.125	1.023 ± 2.384	0.846	0.990	1.243	0.0009	0.0027
***cis*** **-13,16-Docosadienoic acid**	C22:2Δ13,16	38.89	350.58	0.116 ± 0.059	0.084	0.102	0.140	0.144 ± 0.049	0.114	0.129	0.161	0.0014	0.0034
**Arachidonic acid**	C20:4Δ5,8,11,14	38.08	318.49	10.83 ± 4.169	7.929	10.660	13.790	14.43 ± 5.755	12.040	14.930	16.070	0.0008	0.0027
**Tricosanoic Acid**	C23:0	37.42	368.64	0.219 ± 0.154	0.139	0.209	0.336	0.238 ± 3.177	0.011	0.292	0.391	0.5651	0.5651
***cis*** **-11,14,17-Eicosatrienoic acid**	C20:3Δ11,14,17	28.93	320.51	0.684 ± 0.508	0.299	0.527	1.008	1.105 ± 7.267	0.942	1.042	1.267	0.0001	0.0006
**Erucic acid**	C22:1Δ13	35.60	352.59	0.421 ± 0.217	0.276	0.429	0.559	0.633 ± 5.170	0.517	0.636	0.707	0.0001	0.0006
***cis*** **-8,11,14-eicosatreinoic acid**	C20:3Δ8,11,14	37.05	320.51	1.193 ± 0.371	0.990	1.227	1.419	1.211 ± 4.636	0.969	1.176	1.531	0.0535	0.0755
**Behenic acid**	C22:0	35.18	354.61	0.202 ± 0.098	0.142	0.207	0.266	0.258 ± 8.088	0.213	0.255	0.325	0.0001	0.0006
***cis*** **-11,14-eicosadienoic acid**	C20:2 Δ11,14	35.18	308.50	0.687 ± 0.629	0.222	0.429	1.083	0.856 ± 9.508	0.435	0.859	1.369	0.2064	0.2431
**Heneicosanoic acid**	C21:0	34.39	340.58	0.021 ± 0.008	0.015	0.021	0.026	0.023 ± 8.009	0.017	0.024	0.028	0.0312	0.0525
**Linolenic acid**	C18:3Δ9,12,15	34.20	294.26	3.954 ± 1.650	2.722	3.979	4.928	5.195 ± 9.834	4.679	5.519	5.528	0.0001	0.0006
***cis*** **-11-Eicosanoic acid**	C20:1 Δ11	33.45	310.50	1.842 ± 1.316	0.377	1.975	2.640	2.137 ± 2.357	1.123	2.669	2.845	0.1439	0.1919
**Arachidic acid**	C20:0	32.85	326.56	0.224 ± 0.079	0.169	0.239	0.266	0.250 ± 9.088	0.216	0.257	0.287	0.0005	0.0020
**Linoleic acid**	c-C18:2Δ9,12	32.38	294.47	0.641 ± 0.576	0.016	0.743	0.988	0.720 ± 9.747	0.020	0.730	1.283	0.3740	0.3903
**Linolelaidic acid**	t-C18:2Δ9,12	31.29	294.47	0.017 ± 0.010	0.011	0.041	0.019	0.021 ± 1.009	0.013	0.020	0.028	0.2111	0.2413
**Oleic acid**	c-C18:1Δ9	30.82	297.49	26.416 ± 8.524	21.350	28.260	32.480	30.818 ± 8.451	28.110	29.720	31.870	0.0003	0.0014
**Stearic acid**	C18:0	29.79	298.50	22.679 ± 5.695	17.820	23.630	25.710	25.708 ± 6.692	23.420	24.240	25.580	0.0011	0.0029
***cis*** **-10-Heptadecanoic acid**	C17:1Δ10	29.30	284.48	2.932 ± 1.470	1.874	2.621	3.898	3.177 ± 5.925	2.671	2.826	3.444	0.0021	0.0046
**Heptadecanoic acid**	C17:0	28.21	283.48	0.320 ± 0.094	0.248	0.321	0.397	0.375 ± 5.073	0.333	0.366	0.386	0.0448	0.0672
**Palmitoleic acid**	C16:1Δ9	27.77	270.45	0.855 ± 0.409	0.647	0.895	1.098	0.760 ± 1.489	0.289	0.722	1.282	0.1736	0.2193
**Palmitic acid**	C16:0	26.60	270.45	20.953 ± 5.023	17.150	21.220	23.920	22.929 ± 7.392	20.150	22.230	24.660	0.0036	0.0072
**Pentadecanoic acid**	C15:0	24.91	256.42	0.165 ± 0.051	0.133	0.163	0.199	0.187 ± 3.048	0.152	0.173	0.223	0.2345	0.2558

**Table 2:** Fatty acids significantly higher in post-mortem brain tissue from subjects with moderate AD, severe AD, or DLB pathology. Following the Kruskal–Wallis tests, multiple comparison procedures using stepdown permutation *p*-values on the ranked data were used to investigate the nature of group differences for variables with a false discovery rate of 0.05 or less.

Fatty Acid	*p*-Values					
	Control vs. Moderate AD	Control vs. Severe AD	Control vs. DLB	Moderate AD vs. Severe AD	Moderate AD vs. DLB	Severe AD vs. DLB
Nervonic acid (mono)	0.0453	0.7397	0.0453	0.1811	0.9578	0.1811
Erucic acid (mono)	0.0011	1	0.0011	0.0022	1	0.0022
Oleic acid (mono)	0.0006	0.0337	0.0011	0.1912	0.8664	0.1917
*cis*-10-Heptanoic acid (mono)	0.0049	0.0899	0.0068	0.3476	0.8887	0.3476
*cis*-13,16-Docosadienoic acid (poly)	0.0036	0.5416	0.0104	0.0331	0.7307	0.0707
Arachidonic acid (poly)	0.0094	1	0.0094	0.0128	1	0.0128
*cis*-11,14,17-Eicosatrienoic acid (poly)	0.0001	0.4203	0.0001	0.0004	0.9930	0.0004
Linolenic acid (poly)	0.0003	0.6126	0.0004	0.0029	0.8786	0.0039
Lignoceric acid (saturated)	0.0004	0.1263	0.0333	0.0514	0.4317	0.4317
Behenic acid (saturated)	0.0002	0.1151	0.0006	0.0186	0.7104	0.0455
Arachidic acid (saturated)	0.0001	0.3774	0.0783	0.0046	0.2069	0.3774
Stearic acid (saturated)	0.0006	0.2969	0.0344	0.0344	0.3416	0.3416
Palmitic acid (saturated)	0.0065	0.0902	0.0143	0.4114	0.7813	0.4832
Number of FAs significantly different	13	1	12	9	0	5

## Supplementary Materials

The following are available online at http://www.mdpi.com/2218-1989/8/4/69/s1, Table S1: Descriptive characteristics of the study population by group, Table S2: Aggregated fatty acid concentrations and ratios, Table S3: Associations between fatty acids and clinical variables (age, gender, post-mortem delay, frontal tissue pH, beta-amyloid, tau); Figure S1. Principal component analysis (PCA) of fatty acid concentrations in post-mortem human brain specimens.
