# Supplementary material for: Evidence That Parietal Lobe Fatty Acids May Be More Profoundly Affected in Moderate Alzheimer’s Disease (AD) Pathology Than in Severe AD Pathology
Source: Metabolites. 2018 Oct 26;8(4):69. doi: 10.3390/metabo8040069 (PMC6316131; doi:10.3390/metabo8040069)
Supplement: Supplementary file 1 [file metabolites-08-00069-s001.pdf]

Table S1. Descriptive characteristics of the study population by group.

	Control (n=27)	Moderate AD (n=16)	Severe AD (n=30)	DLB (n=14)
**Age (years) ǂ**	80.7 (8.3)	85.6 (8.4)	82.5 (6.2)	78.8 (7.7)
**Female/male, nǂ**	13/13	8/7	15/15	5/9
**Postmortem delay (hours) ǂ**	39.6 (18.4)	30.7 (17.9)*	48.4 (23.4)*	36.9 (19.6)
**Braak Stage, n (0/1/2/3/4/5/6)**	(7/6/14/0/0/0/0)	(0/0/0/5/11/0/0)	(0/0/0/0/0/12/18)	(4/0/6/3/0/0/1)

ǂResults are presented as mean (±SD). Group comparisons of patient characteristics (age, % female and post-mortem delay) were compared by parametric one-way ANOVA. There were no significant differences between groups for age or gender. *Post-mortem delay was significantly longer in cases of severe AD compared with cases of moderate AD (p = 0.03).

Table S2. Aggregated fatty acid concentrations and ratios.

	Control	Moderate AD	Severe AD	DLB	P-value
**Total FA** (g/kg dry weight)	114.7 ± 5.414	148.4 ± 3.612	128.4 ± 5.864	149.9 ± 6.807	<0.001
**Total SFA** (g/kg dry weight)	40.24 ± 1.878	51.19 ± 1.119	45.72 ± 2.052	50.99 ± 2.317	<0.001
**Total MUFA** (g/kg dry weight)	32.32 ± 1.569	43.52 ± 1.724	36.81 ± 1.997	42.87 ± 1.860	<0.001
**Total PUFA** (g/kg dry weight)	42.04 ± 2.222	53.63 ± 1.291	45.82 ± 2.446	56.02 ± 3.205	<0.001
**Omega 3/Omega 6**	2.454 ± 0.654	2.421 ± 0.510	2.730 ± 1.573	2.465 ± 0.567	0.700
**16:1/16:0**	1.126 ± 0.405	1.146 ± 0.469	1.082 ± 0.118	1.121 ± 0.393	0.933

Results are presented as mean (±SD). Aggregated fatty acid concentrations and ratios were derived from individual concentrations (Table 1). P-values were generated from the Kruskal Wallis (KW) test with a P-value of less than 0.05 deemed to be statistically significant.

Table S3. Associations between fatty acids and clinical variables (age, gender, post-mortem delay, frontal tissue pH, beta-amyloid, tau).

**Fatty acid**	**Gender**	**Age**		**Post-mortem delay**		**Frontal tissue pH**		**Beta-amyloid**		**Tau**	
	**P-value**	**Spearman r**	**P-value**	**Spearman r**	**P-value**	**Spearman r**	**P-value**	**Spearman r**	**P-value**	**Spearman r**	**P-value**
**Docosahexanoic Acid**	0.9930	-0.04181	0.6972	-0.0623	0.5642	0.0276	0.887	0.2803	0.1951	-0.424	0.0964
**Nervonic Acid**	0.5844	-0.1096	0.3067	-0.2028	0.0581	-0.02292	0.9061	-0.105	0.6335	-0.201	0.4481
**Lignoceric Acid**	0.5841	-0.1563	0.1436	**-0.315**	**0.0028***	-0.07566	0.6965	0.1778	0.417	-0.4044	0.1063
***Cis*-13,16-Docosadienoic**	0.6424	0.05779	0.5907	-0.1615	0.1328	0.09612	0.6199	0.3749	0.0779	-0.2426	0.3357
**Arachidonic acid**	0.7821	-0.1011	0.3459	**-0.2501**	**0.0188***	-0.105	0.5878	0.1426	0.5162	-0.4632	0.0312
**Tricosanoic Acid**	0.2755	-0.1938	0.0688	-0.1501	0.1627	0.2783	0.1439	0.1788	0.4143	-0.1471	0.5616
***Cis*-11,14,17-Eicosatrienoic**	0.3278	-0.01734	0.8719	**-0.2704**	**0.0108***	-0.1698	0.3785	0.03863	0.8611	**-0.7304**	**0.0013***
**Erucic acid**	0.2273	0.03136	0.7705	-0.2062	0.054	-0.1935	0.3146	0.1783	0.4156	**-0.5735**	**0.0175***
***Cis*-8,11,14-eicosatreinoic**	0.7939	0.02736	0.7991	0.04522	0.6757	0.0971	0.6163	0.3982	0.0598	0.152	0.5601
**Behenic acid**	0.8535	-0.105	0.3276	-0.3132	0.003	-0.09119	0.638	0.1471	0.503	-0.4142	0.0995
***Cis*-11,14-eicosadienoic acid**	0.6927	0.02597	0.8091	-0.03961	0.7141	-0.105	0.5878	0.207	0.3432	-0.3529	0.1621
**Heneicosanoic acid**	0.2348	-0.02369	0.8256	-0.1736	0.1058	-0.0732	0.7059	0.07875	0.721	-0.1569	0.5459
**Linolenic acid**	0.3510	0.03517	0.7435	**-0.2148**	**0.0445***	-0.1538	0.4257	0.1218	0.5797	-0.6642	0.0051
***Cis*-11-eicosanoic acid**	0.7543	0.06844	0.5239	0.03979	0.7128	-0.1481	0.4432	0.2888	0.1815	-0.3676	0.1121
**Arachidic acid**	0.5125	-0.00498	0.9631	**-0.2716**	**0.0105***	-0.07936	0.6824	0.1664	0.4479	-0.4167	0.0998
**Linoleic acid**	**0.0469***	-0.01845	0.8638	-0.09548	0.3762	-0.3571	0.0572	0.1426	0.5162	-0.09804	0.6642
**Linolelaidic acid**	0.3363	0.06029	0.5747	0.06752	0.5319	-0.1866	0.3325	-0.06686	0.7618	**-0.5**	**0.040***
**Oleic acid**	0.5585	-0.05324	0.6202	-0.2015	0.0598	-0.1824	0.3437	0.1456	0.5074	-0.2451	0.3213
**Stearic acid**	0.7881	-0.0168	0.8758	**-0.2421**	**0.023***	-0.05767	0.7663	0.2793	0.1967	-0.2647	0.3003
***Cis*-10-heptadecanoic acid**	0.7840	0.09948	0.3537	-0.2131	0.0463	-0.08404	0.6647	0.3522	0.0994	-0.1544	0.561
**Heptadecanoic acid**	0.9101	-0.09664	0.3676	-0.1261	0.2416	-0.1294	0.5035	0.1506	0.4929	-0.3505	0.1118
**Palmitoleic acid**	0.1840	-0.05153	0.6315	-0.03188	0.7681	-0.05915	0.7605	0.2932	0.1745	-0.201	0.4214
**Palmitic acid**	0.4333	-0.005368	0.9602	-0.09706	0.3683	0.04091	0.8331	0.4131	0.0510	-0.1299	0.6041
**Pentadecanoic acid**	0.0754	-0.07179	0.5038	0.04819	0.6557	-0.1866	0.3325	0.1501	0.4943	-0.451	0.085

P-values for gender were obtained by dividing all cases into either male or female and conducting a Mann-Whitney U test. All other results were obtained by conducting Spearman r correlation analysis on the stated continuous variables. Statistically significant results are indicated with an asterisk.

Figure S1. Principal Component Analysis (PCA) of fatty acid concentrations in post-mortem human brain specimens.

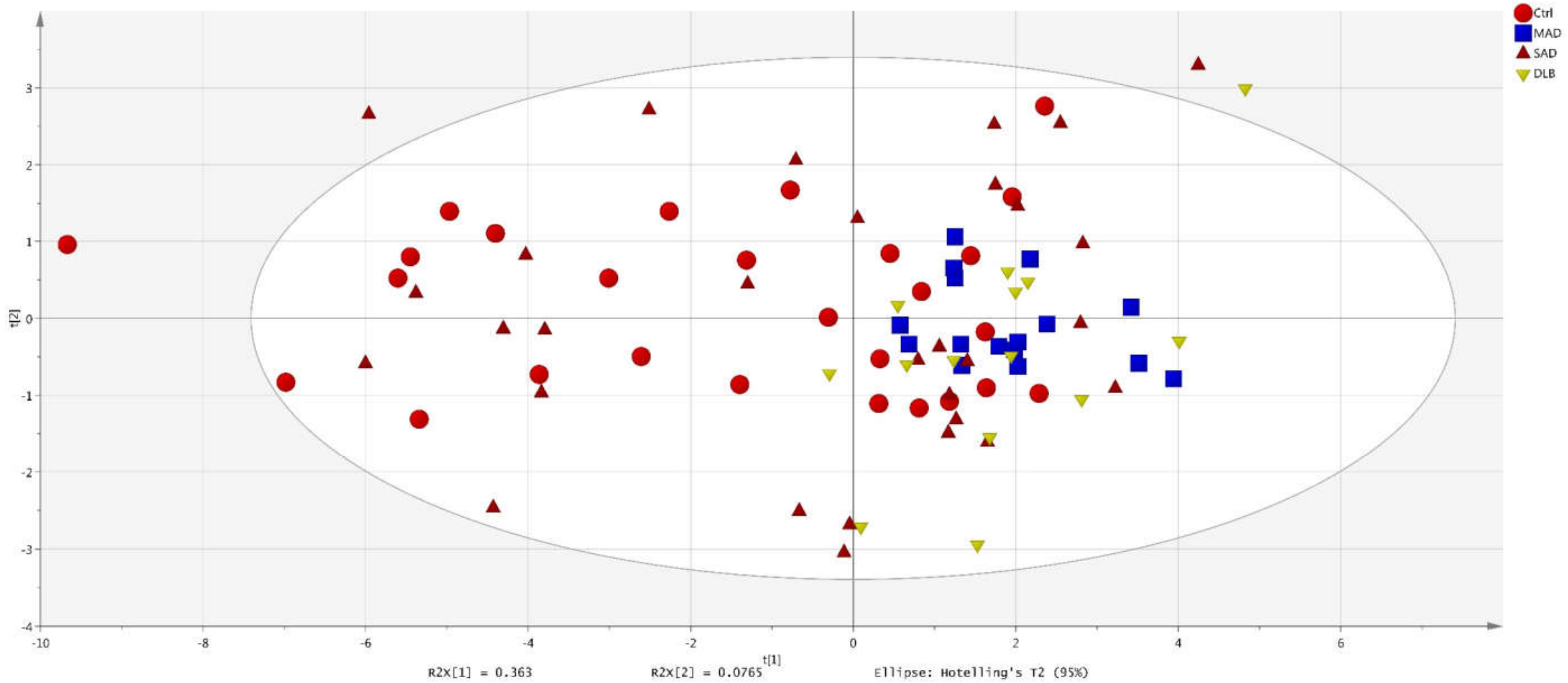

PCA was completed using Simca P (v14.1; Umetrics, Umea, Sweden) based on mean centered and log transformed prior to analysis. The scores plot above was generated by principal component analysis (PCA). The first two components accounted for approximately 43% of the total variance; whereas the first component (PC1) explained 36.3% of the variation, the second component (PC2) explained 7.7%. Furthermore, PC3 showed no distinct group separation pattern. Red circles = Control; Blue Squares = Moderate AD; Red triangles = Severe AD; Yellow triangles = Dementia with Lewy Bodies.
